# Carence en fer, anémie et anémie ferriprive chez les donneurs de sang à Kinshasa, République Démocratique du Congo

**DOI:** 10.11604/pamj.2016.23.174.7662

**Published:** 2016-04-13

**Authors:** Franck Nzengu-Lukusa, Sylvain Yuma-Ramazani, Eddy Sokolua-Mvika, Angèle Dilu-Keti, Blanchard Malenga-Nkanga, Jean Baptiste Shuli, Donatien Kayembe Nzongola-Nkasu, Ferdinand Mbayo-Kalumbu, Steve Ahuka-Mundeke

**Affiliations:** 1 Departement de Biologie Médicale, Cliniques Universitaires de Kinshasa, Kinshasa, RDC; 2 Centre Hospitalier Monkole, Kinshasa, République Démocratique du Congo; 3 Centre National de Transfusion Sanguine, Kinshasa, République Démocratique du Congo; 4 Ministere de la Santé Publique, Kinshasa, RDC; 5 Institut National de Recherche Biomédicale, Kinshasa, République Démocratique du Congo

**Keywords:** Carence en fer, anémie, donneur de sang, Kinshasa, Iron deficiency, anemia, blood donor, Kinshasa

## Abstract

**Introduction:**

En République Démocratique du Congo (RDC), plus d'un million de don de sang ont été réalisés entre 2007 et 2011. Cependant, aucun bilan portant sur la carence en fer et l'anémie ferriprive, conséquence d'un don de sang chez les donneurs de sang (DS), n'est disponible dans ce pays. L'objectif de cette étude était d'estimer la prévalence de la carence en fer, de l'anémie et de l'anémie ferriprive chezles DS au Centre National de Transfusion Sanguine (CNTS) à Kinshasa en RDC.

**Méthodes:**

Entre Décembre 2012 et Août 2013, une étude transversale a été menée au CNTS où des DS éligibles au don de sang ont été inclus. Les informations socio démographiques et des prélèvements sanguins ont été collectés de manière simultanée au don de sang. La ferritine sérique a été dosée pour évaluer la carence en fer en utilisant la technique ELISA. L'hémogramme a été réalisé en vue d’évaluer et mettre au point l'anémie.

**Résultats:**

Au total 386 DS ont été inclus dans cette étude. La prévalence de la carence en fer et de l'anémie ferriprive étaient respectivement de 63,2% (244/386) et 25,9% (100/386) des DS. Une anémie a été trouvée chez 36.5% (141/386) au moment du don de sang.

**Conclusion:**

La carence en fer, l'anémie et l'anémie ferriprive demeurent très fréquentes chez les DS à Kinshasa. Ces résultats suggèrent la révision des tests biologiques utilisés dans le recrutement des DS au CNTS. Par ailleurs le dosage de la ferritine s'impose en routine chez les DS rég

## Abstract (en)

**Introduction:**

In the Democratic Republic of Congo (DRC), the practice of blood transfusion is common with more than two hundred thousand one million blood donation per year has been made between 2007 and 2011. However, no report on iron deficiency and iron deficiency anemia as a result of a donation blood in DS is available in this country. This study aimed to estimate the extend of iron deficiency, anemia and iron deficiency anemia in volunteer blood donors (DS) in the National Blood Transfusion Center (CNTS) in Kinshasa, DRC.

**Method:**

A cross-sectional study was conducted in CNTS where Kinshasa volunteer blood donors were included. Socio demographic information and blood samples were collected. Hematological parameters and Serum ferritin was assessed using respectively standard colorimetric and ELISA techniques.

**Result:**

A total of 386 DS were included in this study. The prevalence of iron deficiency and iron deficiency anemia were respectively 63.2% (244/386) and 25.9% (100/386) of DS. Anemia was found in 36.5% (141/386) at the time of blood donation.

**Conclusion:**

Anemia, iron deficiency and iron-deficiency anemia are highly prevalent among blood donors in Kinshasa, DRC. Hence the needs to review the screening tests for the selection of blood donors and also include serum ferritin measurement for the routine assessment of blood donors, especially among regular blood donors.

## Introduction

La transfusion sanguine constitue une alternative thérapeutique très importante dans la prise en charge des anémies sévères. Cependant, elle peut exposer des conséquences aussi bien chez les donneurs que chez les receveurs de sang. Chez les receveurs, elle peut entraîner les complications d'ordre métabolique, immunoallergique ou infectieuse. Par contre pour les donneurs de sang (DS), ces complications sont essentiellement d'ordre carentiel particulièrement martial. En effet, un don de sang de 450 ml peut occasionner une perte de 213 à 236 mg de fer qui correspond au moins à cent jours d'apports alimentaires en fer [1, 2]. Pour prévenir ces complications chez les DS, il est recommandé de limiter le nombre des dons de sang à 4 par an, associé ou non à la supplémentation en fer chez les DS réguliers dans certaines régions [2]. Cependant, dans beaucoup des pays, particulièrement en développement ces normes ne sont généralement pas respectées [2–4]. La conséquence en est qu'un DS régulier s'expose à une carence martiale qui peut évoluer vers une anémie ferriprive [2, 3]. Celle-ci évolue en trois phases: d'abord une diminution de la ferritine sérique puis du fer sérique, puis une diminution du taux d'hémoglobine et enfin des modifications cytologiques traduites par une diminution des constantes hématimétriques [2, 4]. Ainsi, les taux d'hémoglobine et d'hématocrite, fréquemment utilisés comme paramètres biologiques de recrutement de DS dans la politique transfusionnelle, restent insuffisants pour détecter la carence en fer chez les DS [5]. En République Démocratique du Congo (RDC), la pratique de la transfusion est très importante avec plus d'un million de dons de sang réalisé entre 2007 à 2011. Cependant le taux de fidélisation de DS reste encore faible [3]. De plus, la limitation de 4 dons de sang par an pour chaque DS n'est pas systématiquement respectée et la supplémentation en Fer chez les des DS réguliers non recommandée. Le taux d'hémoglobine et/ou d'hématocrite reste les seuls critères biologiques d'inclusion de DS. Cependant, à notre connaissance, aucune étude évaluant la carence en fer, l'anémie et l'anémie ferriprive chez les donneurs de sang n'a été réalisée en RDC, particulièrement à Kinshasa où plus du quart des dons de sang de la RDC sont réalisés [4]. L'objectif de ce travail était de déterminer la prévalence de la carence en fer, de l'anémie et de l'anémie ferriprive chez les DS bénévoles à Kinshasa en RDC et d'en identifier les facteurs associés.

## Méthodes

Il s'agit d'une étude transversale, descriptive et analytique réalisée de Décembre 2012 à Août 2013 à partir des DS bénévoles recrutés au CNTS ou lors des campagnes de collectes mobiles organisées par le CNTS à travers différents sites à Kinshasa. Nous avons inclus ceux qui étaient consentants et considérés comme éligibles au don de sang par le CNTS durant la période de l’étude [5]. Chaque DS retenu a fait l'objet d'une étude portant sur le sexe, l’âge, la profession, le nombre total de don de sang antérieur, le nombre de don de sang au cours des 12 derniers mois précédant le don actuel, ainsi que l'intervalle de temps entre le don de sang actuel et le dernier. De plus, pour chaque donneur, deux prélèvements de sang ont été réalisés simultanément. L'un de 10 ml s'est fait sur tube sec, et l'autre de 5ml sur tube avec anticoagulant (EDTA). Le premier a servi de réaliser un hémogramme complet au laboratoire du centre national de transfusion sanguine à l'aide d'un automate SYSMEX KX 21 N (Systmex America Corporation, USA), et le deuxième le dosage de la ferritine sérique par méthode d'ELISA (kit “Ferritin de Diamétra^®^”, Italie). La lecture était faite à une longueur d'onde de 450 nm contre le blanc. La carence en fer a été définie par un taux de ferritine <20ng/mL pour l'homme et <8ng/mL pour la femme. La présence simultanée d'anémie et de carence en fer a été considérée comme anémie ferriprive. Les variables en rapport avec les caractéristiques du don de sang ont fait l'objet d'un codage: le nombre total de don a été regroupé en 4 groupes: 1, 2 à 5, 6 à 10 et plus de 10 dons; l'intervalle de deux derniers dons regroupé en 3 classes: aucun (premier don), 3 mois et plus de 3 mois; le nombre de dons des 12 mois précédents l'inclusion regroupée en 2 classes: de 0 à 4 dons et plus de 4 dons. Les données sociodémographiques et biologiques ont été saisies sur le logiciel Excel 2007 et l'analyse statistique a été réalisée à l'aide du logiciel SAS version 9.3 (SAS Institut Inc., USA) au seuil de significativité de 5%. La régression logistique multivariée a été réalisée pour étudier la relation entre l'anémie, la carence en fer, l'anémie ferriprive et les différents facteurs d'intérêt. La sélection des variables a été réalisée par une analyse univariée au seuil de significativité de 20%, puis celles qui étaient significatives ont été retenues pour le modèle final au seuil de significativité de 5%. Certaines variables d'ajustement ont été forcées dans le modèles. Les odds ratio ajustés ont été calculés avec leur intervalle de confiance à 95%.

## Résultats

Au cours de la période d’étude, 386 DS ont été inclus pour cette étude. Il s'agissait majoritairement (80,6%) des hommes avec sex-ratio H/F de 4/1. L’âge moyen était de 36 ans (extrêmes: 18 et 66 ans). Sur les 385 DS, 64(16,58%) faisaient pour la première fois leur don, alors que 30,31%(117/386) étaient entre 2 et 5 dons, 12,18% (47/386) entre 6 et 10 dons et 40,93% (158/386) étaient à plus de 10 dons. Parmi les DS retenus 17,96% (67/386) d'entr'eux n'avaient pas réalisé des dons au cours de 12 derniers mois tandis que 22,25% (83/386) étaient à plus de 4 dons au cours de la même période. Par ailleurs 66,8% des DS avaient 3 mois comme intervalle de temps entre le don d'inclusion et celui qui précédait par contre 12,5% (46/386) avaient un délai > 3 mois (Tableau 1). Le Tableau 2 montre que la prévalence de la carence en fer était de 63,2% (244/386), celle de l'anémie était de 36,5% (141/386) et l'anémie ferriprive a été retrouvée chez 25,9% (100/386) de DS. Chez les hommes, la prévalence de la carence en fer était de 70,42% (219/311), celle de l'anémie était de 31,9% (97/311) et celle de l'anémie ferriprive était à 26,05% (81/311) tandis chez les femmes, des prévalences respectives de 33,33% (25/75), de 58,67 (44/75) et 25,33% (19/75) ont été retrouvées. Enfin la carence en fer était estimée à 42,19% (27/64), l'anémie à 23% (15/64) et l'anémie ferriprive à 14,06% (9/64) chez les DS au premier don. Pour les DS ayant réalisé 2 à 5 dons, les prévalences à la carence en fer, anémie et de l'anémie ferriprive étaient respectivement estimées à 52,99% (62/117); 34,19 (40/117) et 17,95% (40/117). Pour ceux au delà 10 dons, les prévalences de 77,85% (123/158) de carence en fer, de 41,77(66/158) d'anémie et 32,91% (52/158) d'anémie ferriprive ont été estimées chez les DS. Le Tableau 3 illustre la relation entre l'anémie, la carence en fer, l'anémie ferriprive et les différents facteurs. il ressort quel'anémie était indépendante des caractéristiques du don de sang (le nombre total de don au cours de la vie, l'intervalle entre les deux derniers dons et le nombre de don des 12 mois précédents l'inclusion). Par contre elle reste liée au sexe, avec une plus grande susceptibilité des femmes de faire l'anémie par rapport aux hommes (ORa = 0.3 [0.1-0.5]; p <0.0002). D'autres part, nous avons observé un lien statistiquement significatif entre le sexe (p <0.0001), le nombre total de don au cours de la vie (p =0.02), l'intervalle de temps entre les 2 derniers dons (p =0.008) et la carence en fer. Le sexe masculin est plus à risque de faire une carence en fer (ORa = 6,0 [3,0-11,5]. Un nombre total de don supérieur à 10 dons est plus à risque qu'un total de 2 à 5 dons (ORa = 2,3[1,2-4,4]). De plus un intervalle entre les deux derniers dons supérieur à 3 mois est un facteur protecteur de la carence en fer par rapport à un intervalle de 3 mois (ORa = 0,3 [0,1-0,7]).

**Tableau 1:** Les principales caractéristiques sociodémographiques de la population d’étude

Caractéristiques	Fréquence (N = 386)	%
**Sexe**		
Masculin	311	80,6
Féminin	75	19,4
**Age (ans)**		
<30	126	32,64
30-39	109	28,24
40-49	89	23,06
>50	62	16,06
**Etat civil**		
Célibataire	206	53,8
Marié	166	43,3
Divorcé	4	1,04
Veuf	7	1,8
**Province d'origine**		
Bas-Congo	164	42,4
Bandundu	66	17,1
Equateur	52	13,4
Kasaï Occidental	10	2,59
Kasaï Oriental	55	14,2
Katanga	6	1,5
Maniema	9	2,3
Nord-Kivu	4	1
Province Orientale	17	4,4
Sud-Kivu	3	0,72
**Religion**		
Catholique	141	37,8
Protestant	139	37,2
Réveil	73	19,5
Kimbanguiste	9	2,4
Autre	11	2,9

**Tableau 2:** Les prévalences de la carence en fer et anémie ferriprive des donneurs de sang de la population d’étude

Catégorie	Prévalence (%)		
	Carence en fer	Anémie	Anémie ferriprive
**Population d’étude**			
	**63,2** (244)	**36,5** (141)	**25,9** (100)
**Sexe**			
Masculin	**70,42** (219)	**31,19** (97)	**26,05** (81/311)
Féminin	**33,33** (25)	**58,67** (44)	**25,33** (19/75)
**Age**			
<30	**53,97** (68/126)	**31,75** (40/126)	**19,05** (24/126)
30-39	**67,89** (74/109)	**36,7** (40/109)	**26,61**(29/109)
40-49	**71,91** (64/89)	**42,7** (38/89)	**34,83** (31/89)
>50	**61,29** (38)	**37,1** (23/62)	**25,81**(16/62)
**Nombre total des dons**			
1	**42,19** (27/64)	**23,44** (15/64)	**14,06** (9/64)
2 à 5	**52,99** (62/117)	**34,19** (40/117)	**17,95** (21/117)
6 à 10	**68,09** (32/47)	**42,55** (20/47)	**38,3** (18/47)
>10	**77,85** (123/158)	**41,77** (66/158)	**32,91**(52/158)

**Tableau 3:** Les principaux facteurs associés à la carence enf et à l'anémie ferriprive de la population d’étude

	Anémie		Carence en fer		Anémie ferriprive	
	OR (IC95%)	ORa (IC95%)	OR (IC95%)	ORa(IC95%)	OR (IC95%)	ORa (IC95%)
**Age (année)**						
p-value	0,75 0,99 (0,9-,04)		0,46 1,0 (0,9-1,06)		0,24 1,03 (0,1-1,1)	
**Sexe** *p-value* M vs F	*0,000* 0,31 (0,1-0,5)	*<0,0002* 0,3 (0,1-0,5)	*0,000* 3,57(2,1-6,9)	*<0,0001* 6,0 (3,0-1,5)	0,35 0,75 (0,4-1,3)	*0,96* 1,0 (0,5-2,0)
**Nombre total de don** *p-value* 6-10 vs 2-5 >10 vs 2-5	0,38 1,4 (0,7-2,8) 1,3 (0,8-2,2)	0,28 1,4 (0,6-3,1) 1,6 (0,9-2,8)	*0,000* 1,88 (0,9-3,8) 2,9 (1,7-4,9)	*0,02* 1,3 (0,6-3,1) 2,3 (1,2-4,4)	*0,025* 2,4 (1,1-5,0) 1,9 (1,1-3,4)	*0,03* 2,4(1,08-5,4) 2,1 (1,1-4,0)
**Délai des 2 derniers dons (mois)** *p-value* >3 vs 3	0,36 0,76 (0,4-1,3)	0,97 0,9 (0,4-1,9)	*0,000* 0,36(0,2-0,5)	0,008 0,3 (0,1-0,7)	*0,16* 0,62 (0,3-1,2)	0,98 0,9 (0,4-2,1)
**Nombre de dons des 12 mois précédents** p-value 0-4 vs >4	0,67 0,89 (0,5-1,4)	0,49 1,2 (0,6-2,3)	*0,000* 0,45(0,2-0,7)	0,35 0,7 (0,3-1,4)	0,27 0,79 (0,4-1,2)	0,89 0,9 (0,5-1,7)

## Discussion

Cette étude qui rapporte les résultats observés chez les DS bénévoles du CNTS aurait pu être élargie aux centres de transfusion sanguine des autres hôpitaux et centres de santé de la ville de Kinshasa. Ce qui permettrait d'extrapoler les résultats à toute la population de Kinshasa. D'autre part, elle n'a pas inclus les DS familiaux et rémunérés qui représentent plus de 60% de DS à Kinshasa [3]. Ce qui réduit une fois de plus l'intérêt épidémiologique de l’étude. Toutefois à notre connaissance, c'est la première étude qui rapporte des informations sur la carence en fer, l'anémie et l'anémie ferriprive chez les DS bénévoles en RDC. Elle donne un aperçu sur le problème que pose le don de sang dans les pays en développement en terme de conséquences chez les DS. Ces résultats pourraient être utilisés par le CNTS dans l'optique de changer les conditions de recrutement des DS. Ce qui permettrait de minimiser les troubles nutritionnels déjà fréquents au sein de la population dans les pays en développement. Dans cette étude, il ressort que 63,2% de DS bénévoles présentaient une carence en fer, 36,5% étaient anémiques et chez 25%, l'anémie ferriprive. Il apparaît également que la carence en fer était associée au sexe, au nombre total de don de sang ainsi qu’à l'intervalle entre les deux derniers dons. L'anémie seule n’était associée qu'avec le sexe alors que l'anémie ferriprive n’était associée qu’ au nombre total des dons. La prévalence de la carence en fer rapportée dans cette étude semble très élevée que celles de Jeremiah au Nigeria, 20,6% [2] et de Boulahrissau au Maroc, 43% [6]. La divergence de résultats s'explique par des différences d'ordre méthodologique. En effet, Jeremiah et coll ont inclus 3 types de DS (familiaux, bénévoles et rémunérés) ayant au moins 10 g/dl d'hémoglobine et le seuil de 12µg/L de ferritine sérique a été fixé pour définir la carence en fer (sans tenir compte du sexe ni de l’âge des DS). Boulahriss et coll par contre n'ont inclus que des DS de sexe féminin répartis en DS réguliers et ceux au premier don [6]. Dans cette étude, la prévalence de la carence en fer augmentait avec le nombre de don de sang. Cette observation rejoint celle de Lee et coll [7] à Hong Kong qui ont rapporté une prévalence de la carence en fer de 0% au premier don et 35,1% chez les DS réguliers pour les hommes, alors que pour les femmes, ces chiffres étaient respectivement de 7,2% et 65%. De même câble et coll [8] dans une étude américaine regroupant les DS au premier don et DS réguliers confirment cette tendance avec une prévalence presque dix fois plus élevée chez les DS avec plus de 10 dons (31%) comparés à celle au premier don (3,6%). La prévalence de l'anémie et celle de l'anémie ferriprive rapportées dans cette étude sont très élevées que celle rapportée au Nigeria par Jeremiah [3]. Comme pour la carence en fer cette différence peut s'expliquer par le type et le critère d'inclusion de DS considérés ainsi que la valeur seuil utilisée dans la définition de la carence en fer dans chacune de ces études. La forte prévalence de l'anémie en RDC constitue une autre explication. En effet, l'enquête démographique et sanitaire (EDS) réalisée en 2007 en RDC avait montré une prévalence de l'anémie respectivement 53% et 20% chez les femmes et les hommes âgés de 15-49 ans [9]. La prévalence de l'anémie et de l'anémie ferriprive dans cette étude augmentent avec le nombre total de dons. Cette tendance a été également décrite par les auteurs en Afrique et à ailleurs. En effet Javadzadeh et coll. [10] ont trouvé une prévalence de 26,5% chez les DS réguliers alors qu'il n'en avait pas trouvé y avait pas des DS au premier don. De même Goldman [11] retrouve également l'augmentation de la fréquence de l'anémie entre les nouveaux donneurs et ceux ayant >2 dons. Chez les femmes elle passe de 3,8 à 10.2% pendant que chez les hommes de 0,2 à 0,8%. Enfin Hannah et coll. [12] en Australie ont confirmé la présence de la carence en fer chez les nouveaux donneurs de l'ordre de 12% et 1,3% respectivement chez les femmes et les hommes. Cette carence martiale était de 26,4% chez les femmes ayant l'antécédent d'au moins un don au cours de l'année passée. Dans cette population des DS féminins, la carence augmentait avec les nombres des dons et diminuait avec l’âge. Chez les hommes de cette catégorie la carence était de 6,3% sans influence du nombre de dons ni de l’âge du donneur.

## Conclusion

La carence en fer, l'anémie et l'anémie ferriprive demeurent très fréquentes chez les DS à Kinshasa. Cette fréquence est plus élevée chez la femme que chez l'homme et augmente avec le nombre de dons de sang. Ces résultats suggèrent la révision des tests biologiques utilisés dans le recrutement des DS au CNTS. Par ailleurs le dosage de la ferritine en routine chez les DS réguliers s'impose afin de réduire la carence en fer, qui représente le déficit nutritionnel le plus fréquent au monde, particulièrement dans les pays en développement.

### Etat des connaissances sur le sujet

- Plusieurs études ont montré que le don de sang régulier entraîne la carence martiale chez les donneurs de sang. Cette carence évolue avec le nombre de dons de sang réalisé au cours de la vie.

### Contribution de notre étude a la connaissance

- Cette étude apporte des informations pertinentes sur l'ampleur de la carence martiale, de l'anémie et l'anémie ferriprive chez les donneurs de sang bénévoles à Kinshasa, la capitale de la RDC.
- Elle suggère par ailleurs le non respect des critères de recrutement de donneurs de sang à Kinshasa.
